# A review of the long-term use of proton pump inhibitors and risk of celiac disease in the context of HLA-DQ2 and HLA-DQ8 genetic predisposition

**DOI:** 10.1097/MD.0000000000035351

**Published:** 2023-09-22

**Authors:** Alexandra McMillan, Christopher Perez, Amanda E. Brooks

**Affiliations:** a Rocky Vista University, Parker, CO, USA.

**Keywords:** celiac disease, HLA-DQ2, HLA-DQ8, proton pump inhibitors

## Abstract

Proton pump inhibitors (PPIs) are among the most prescribed and widely used medications; however, the long-term effects of these medications are only beginning to be investigated. Since the introduction of omeprazole in 1989, PPIs have become the first-choice treatment for esophagitis, peptic ulcer disease, Zoster–Ellison syndrome, dyspepsia, and the prevention of ulcers with non-steroidal anti-inflammatory drugs. Recent studies have specifically examined the rise in celiac disease (CD) in this context. This review explores how PPIs may impact the development of CD and highlights the need for additional research into the environmental and genetic factors that influence the development and progression of the disease. A literature search was performed using the keywords celiac disease, proton pump inhibitors, human leukocyte antigen (HLA)-DQ2, HLA-DQ8. The pathogenesis of CD is multifactorial, and human leukocyte antigens are one factor that may contribute to its development. Additionally, pharmaceuticals, such as PPIs, that cause gut dysbiosis have been linked to the inflammatory response present in CD. Recent studies have suggested that the rise in CD could be attributed to changes in the gut microbiome, highlighting the significant role that gut microbiota is proposed to play in CD pathogenesis. Although PPI therapy is helpful in reducing acid production in gastroesophageal disorders, additional information is needed to determine whether PPIs are still an appropriate treatment option with the possibility of developing CD in the future, particularly in the context of HLA-DQ2 and HLA-DQ8 predispositions. This review emphasizes the importance of personalized medicine for individuals with gastroesophageal disorders that require long-term use of PPIs.

## 1. Introduction

Although the pharmacokinetics and pharmacodynamics of proton pump inhibitors (PPIs) are well defined, the side effects of their long-term use in treating various gastroesophageal diseases remain unclear. Placing these side effects in the context of the pathogenesis and treatment of celiac disease (CD), which is relatively well known, hints at the contribution that long-term use of PPIs may play a role in the development of esophageal, gastric, and other intestinal diseases.^[1]^ While it is clear that PPIs have a significant impact on the gut microbiota and consequently on the development and progression of multiple gastrointestinal pathologies, their impact in the context of host genetics remains relatively unknown. Research focused on the standard long-term management of various gastroesophageal disorders with PPIs, particularly in individuals with a genetic background that may predispose them to CD, is scant at best; however, accumulating evidence in the literature suggests that in individuals with HLA-DQ2 or HLA-DQ8 genotypes, long-term use of PPIs may initiate or alter the pathogenesis of CD.^[2]^ The importance of establishing this connection between the development of CD and the use of PPIs cannot be understated.

## 2. Methods

A literature search was performed using the keywords celiac disease, PPI, HLA-DQ2, HLA-DQ8.

Ethics statement: Ethical approval not necessary as the narrative review did not require patient consent.

## 3. Discussion

### 3.1. Proton pump inhibitors

**Figure 1.**
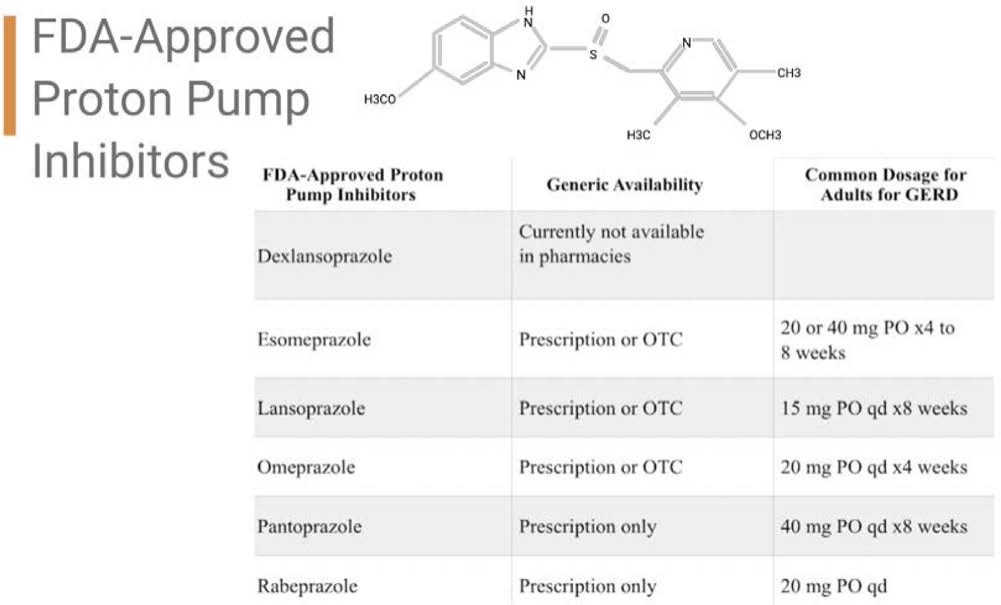
Created with BioRender.com. There are 7 FDA-approved proton pump inhibitors; however, only 6 are available in pharmacies. The table was assembled using www.GoodRx.com.^[5]^

PPIs are commonly prescribed and widely used in the treatment of gastroesophageal diseases to suppress gastric acid secretion.^[3]^ PPIs have gained widespread adoption since their introduction into clinical practice roughly 25 years ago with the development of omeprazole (1988) and lansoprazole (1991) as effective therapies for peptic ulcer disease and gastroesophageal reflux.^[4]^ Currently, there are 7 FDA-approved PPIs that have similar mechanisms, blocking parietal cell acid secretion, but differ in dosage, indication and generic availability (Fig. 1).^[6,7]^ Omeprazole, one of the most commonly used PPIs, was first approved in 2003 for over-the-counter use.^[8]^ The use of PPIs increased by 450% in the 1990s compared to previous years.^[9]^ Today, PPIs continue to be one of the highest-selling classes of drugs with a total of 14 billion spent per year in the United States and 7 billion spent per year globally.^[9]^ The prevalence of PPIs use has continued to increase since the early 2000s with a jump from 9% to 16.8% for omeprazole alone.^[10]^ PPIs bind parietal cell H+/K + ATPases and inactivate them until additional H+/K + ATPases are inserted into the apical membrane of the parietal cells.^[4]^ Unlike other anti-acid drugs, PPIs temporarily halt the end pathway of gastric acid production until compensatory mechanisms resume; therefore, they have excellent benefits when prescribed appropriately.^[11]^ Acid inhibition exceeds the half-life of PPIs, which is around 90 minutes, due to the time necessary for the H+/K + ATPases biosynthesis, roughly 54 hours.^[12]^ Despite their efficacy and their wide availability as both a prescription and over the counter medication, the adverse effects of long-term use of PPIs in the development of various deficiencies and diseases is lacking definitive research.^[13]^ Current observational studies on PPIs suggest association with an increased risk of enteric infections, reduced intestinal absorption of vitamin B12, kidney damage, CD and dementia, revealing serious and significant potential problems with long-term PPI use; however, more controlled clinical trial evidence is needed to confirm not only these associations, but more importantly, identify any causative links.^[14]^

Despite the widespread use of PPIs for various gastric acid-related diseases and the need for more research on their long-term effects, it is clear that some patients develop adverse events related to long-term PPI treatment due to genotypic variability of Cytochrome P450 2C19 (CYP2C19), which is responsible for PPI metabolism.^[12,15]^ Cytochrome P450 is a superfamily of enzymes that oxidize and metabolize a wide variety of therapeutic drugs.^[16,17]^ CYP2C19 has a number of isotypes that are important to consider not only when optimizing PPI efficacy, but also when considering long-term PPI effects.^[18]^ Patients who have a rapid metabolizer genotype of CYP2C19 experience PPI refractory gastroesophageal reflux disease, defined as the persistence of symptoms despite appropriate treatment with PPIs.^[19]^ These findings highlight the importance of assessing PPI therapy carefully in the context pharmacogenetics, where the genotype variability of the CYP2C19 isoenzyme impacts therapeutic efficacy and outcomes, to guide treatment plans as well as patient preference.^[12,19,20]^ CYP2C19 may not be the only important protein to consider in the long-term use of PPIs, HLA (human leukocyte antigens, important markers for immune cell recognition) subtype may also be particularly important in the potential development of CD after long-term PPI therapy.^[2]^

Owing to their initial effectiveness as clinical therapies, PPIs are often the first line of treatment for any gastrointestinal distress, without much consideration of the long-term consequences of their use. This perception has led to recent data showing that PPIs are used in excessive doses and durations, and without clear indications.^[13]^ Nevertheless, there are certain disease indications that necessitate long-term PPI usage. PPIs are crucial for patients with Barrett esophagus (BE) and for the treatment of other acid-related diseases such as gastroesophageal reflux and gastric ulcers.^[20]^ BE is a metaplastic alteration of the lining of the distal esophagus, where esophageal squamous epithelium is replaced by intestinal columnar epithelium, a process thought to be mediated by exposure to acid.^[21]^ Similar to CD, BE has continued to increase in prevalence over the past decade, with an average age of diagnosis of 55 years, with risk factors such as gastroesophageal reflux, *Helicobacter pylori* elimination with acid-suppressive therapy, obesity, tobacco use, and male sex all being hypothesized to influence the development of BE and progression to esophageal adenocarcinoma.^[22,23]^ PPIs decrease acid exposure in the distal esophagus and help to slow the progression of dysplasia in BE.^[4,20]^ However, it is debatable whether PPIs have a negative impact or significant effect on the esophageal microbiome. In a recent study conducted on 58 patients, mucosal biopsies of the distal esophagus were analyzed using a customized esophageal microbiome qPCR panel array, indicating that PPI use does not cause significant changes in the esophageal microbial community.^[24]^ This is counterintuitive to other research on the impact of pharmaceuticals on the gut microbiome, and the small sample size warrants further testing to confirm the effects of PPI use on the esophageal microbiome.^[24]^ Conversely, another study utilizing 16S rRNA gene pyrosequencing concluded that PPI treatment significantly alters gastric and esophageal microbial populations in patients with BE and esophagitis.^[25]^ The paradox between these 2 studies could be attributed to the limitations discussed in the qPCR panel array study, which lacked pH testing to determine the effectiveness of PPI use in individual patients. This extensive testing could have also provided additional data on esophageal microbial composition based on pH.^[24]^ It may also reflect a fundamental difference in the techniques used to detect rarified taxa.

Although the effect of PPIs on esophageal microbiota may not be conclusive, the effects of PPIs on the gut microbiome are clear. Recent studies have highlighted the adverse effects of long-term use of these drugs on the gut microbiome. In the stomach where *H pylori* is abundant, PPIs can cause PPI-induced gastric bacterial overgrowth.^[26]^ Individuals taking PPIs will experience greater pH changes and are subsequently more prone to further overgrowth of *Streptococcus, Lactobacillus,* and other bacteria, leading to impaired bile acid creation, bloating, nausea, and diarrhea.^[27]^ In the proximal duodenum, PPI users have been shown to have an increase in the variation and quantity of the microbiome.^[28]^ A 2010 study of 450 patients found that 50% of PPI users were positive for small intestinal bacterial overgrowth (SIBO) using glucose hydrogen breath tests as opposed to 6% of non-users.^[29]^ A 2012 study using the duodenal aspirates of 300 patients concluded that 36% of PPI users were found to have SIBO as opposed to 22% of non-users.^[4]^ Other than gastric symptomology including bloating and flatulence, SIBO has been associated with iron and vitamin B12 deficiency because of competitive microbial uptake as well as fat malabsorption from the impaired conjugation of bile acids.^[30]^

**Figure 2.**
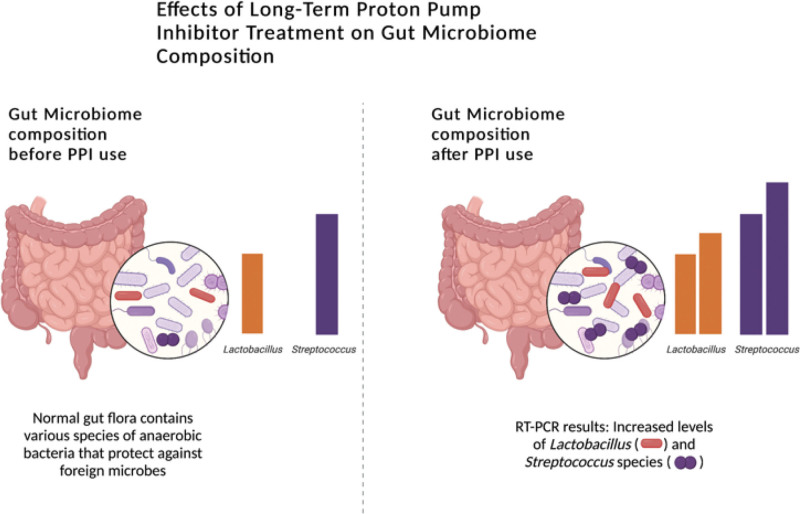
Created with BioRender.com. Long term proton pump inhibitor treatment increases numbers of *Lactobacillus spp*. as well as *Streptococcus spp.* The resulting gut dysbiosis may cause future infectious and inflammatory adverse effects.^[11,33]^

The dysbiosis caused by PPIs may also suggest a potential PPI-celiac disease relationship, as the use of PPIs has also corresponded to a rise in the incidence of celiac disease diagnosis, particularly in children, in recent decades.^[2]^ A population-based control study of 2014 found that those with a celiac disease diagnosis were more likely to have been prescribed PPIs prior to their diagnosis.^[31]^ In the colon, the presence of altered bacterial composition that may predispose one to *C difficile* infection may be the result of PPI-induced overgrowth in the proximal gut.^[32]^ Colonic mucosal inflammation is also suggested in PPI users in which distal gut bacteria can interact with colonic epithelial cells causing the increase of intraepithelial leukocytes.^[4]^ The gut microbiome functions in host resistance against exogenous enteric microbes and overgrowth of commensal microflora.^[11]^ A recent study using quantitative RT-PCR, revealed PPI use was associated with gut dysbiosis.^[11]^ Specifically, there was a significant increase in numbers of *Lactobacillus spp*. Post-PPI treatment, as well as *Streptococcus spp.,* potentially indicating that bacteria commonly present in the throat, nasal, and oral cavities were translocated and were now present in abundance in the intestines (Fig. 2).^[11]^

The concept of bacterial translocation is important when considering the long-term effects of PPIs and the weakened barrier functions that result from decreased levels of gastric acid.^[26]^ Translocation is the process by which bacteria from the gastrointestinal tract progress into other organs and regions of the body by various mechanisms.^[34]^ Pathogen associated molecular patterns from these bacteria are then recognized by pattern recognition receptors of the immune system, which can initiate several signaling pathways as well as carcinogenesis.^[35]^ Distant metastasis from the gut has been shown in patients with colorectal cancer who display high serum levels of calcitonin, which is associated with bacterial infections.^[36]^ This procalcitonin is also present in high levels of pancreatic cancer patients, indicating the presence of bacteria that have translocated from the gut.^[37]^ A study conducted with 300 patients to investigate the relationship between long-term PPI use and *H pylori* infection concluded chronic PPI exposure masks *H pylori* infections, leading to an under-diagnosis of *H pylori* gastritis, as well as increased intestinal metaplasia.^[38]^ One hypothesis by which PPIs mask *H pylori* is through the dilation of oxyntic glands within the stomach causing the protrusion of the parietal cells within the gland and the masking of organisms at the gastric surface or their upregulation in different regions of the stomach.^[38]^ Besides the direct antimicrobial effects of PPIs, another hypothesis is that the increase of gastric pH resulting from the usage of PPIs creates an unfavorable environment for *H pylori*.^[38]^ Interestingly, *H pylori* may exhibit a protective effect in patients with CD, as evidenced by the rise in CD and a correlating decrease in the number of *H pylori* infections.^[39]^ Although this is not a direct indication of causation, the decrease of *H pylori* infections with PPI consumption could be a potential mechanism behind an increased risk of CD with chronic PPI use; however, this hypothesis warrants further research.^[4,39]^

### 3.2. Celiac disease

CD is an autoimmune disease with a strong genetic component that has become more prevalent over the past few decades.^[40]^ Due of the continuously changing nature of the disease. Novel genetic findings, clinical manifestations, and therapeutic strategies are still being discovered.^[41]^ CD was initially described in children; however, the disease can be diagnosed at any age, with adult prevalence increasing in recent years.^[42]^ Current observations regarding the rise in CD and the number of individuals on long-term PPI therapy further underscore the complex nature of CD pathogenesis and the need to fully tease apart the underlying molecular mechanisms of the disease.

While the underlying molecular mechanisms of CD are complex, they are relatively well understood, apart from a few gaps surrounding the interaction of molecular and environmental factors. CD is a T cell-mediated autoimmune disorder caused by gluten proteins with both major histocompatibility complex (MHC) and non-MHC genes included in the list of predisposing genetic factors.^[43,44]^ MHC is a group of genes that code for cell surface proteins that function in the adaptive immune system.^[45]^ HLA genes are proteins that express their gene products on the surface of leukocytes and help regulate the immune system.^[45]^ The MHC region includes HLA genes, which are further subdivided into 3 regions that contain highly polymorphic genes. The class II region, which includes HLA-DQ2 and HLA-DQ8, seems to be critically important in CD development.^[45]^ In fact, the complex immune response elicited in CD involves HLA-DQ2 and HLA-DQ8 restricted T-cells responding to gluten ingestion.^[46]^

**Figure 3.**
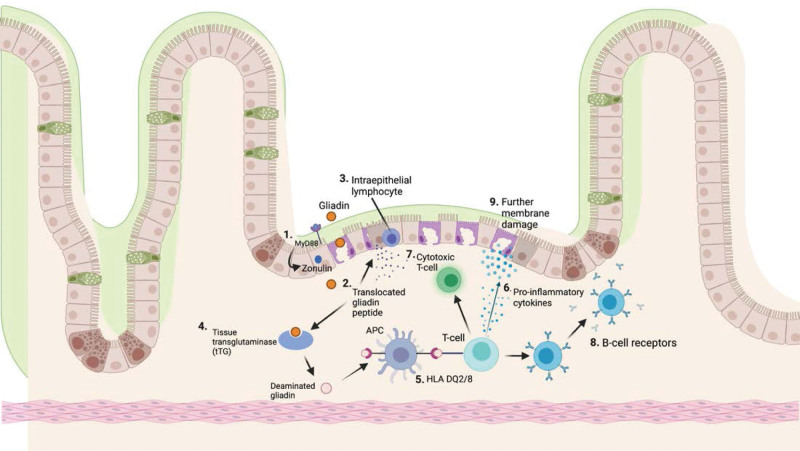
Created with BioRender.com. Celiac disease pathogenesis.^[50]^ Myeloid differentiation factor 88 (MyD88)-dependent zonulin release increases intestinal permeability (Step 1). Gliadin peptide is translocated across intestinal barrier (Step 2). Gliadin peptide causes cytokine release and damage of enterocytes by intraepithelial lymphocytes (Step 3). Tissue transglutaminase (tTG) is released from damaged cells and deaminates gliadin (Step 4). Human leukocyte antigen (HLA) DQ2/8 presents deaminated gliadin to CD4 + T-cell (Step 5). Pro-inflammatory cytokines are released from the activated CD4 + T-cells (Step 6). CD4 + T-cell activation spurs cytotoxic T-cell activation (Step 7). B-cell receptors are activated from CD4 + T-cells, producing anti-tTG and anti-gliadin antibodies (Step 8) that cause further membrane damage and permeability (Step 9). CD = celiac disease, TG = tissue transglutaminase.

Studies have confirmed gliadin, a class of stimulatory proteins that are present in gluten, increase small intestinal permeability through myeloid differentiation factor 88-dependent zonulin release, a proposed factor in the pathogenesis of CD.^[47]^ Myeloid differentiation factor 88 is an adaptor protein for toll-like receptors/IL-1 receptor signaling, which activates inflammatory pathways.^[48]^ Once released zonulin, a protein that modulates permeability of tight junctions between cells of the small intestine, stimulates an innate immune interaction between gliadin and macrophages, an antigen-specific adaptive immune response mediated by T-cells and antigen-presenting cells follows.^[49]^ After zonulin interacts with intestinal epithelium, decreasing the barrier function, the gliadin peptide is able to translocate into the lamina propria, ultimately simulating the release of pro-inflammatory cytokines.^[50]^ Following the subsequent apoptosis of intestinal cells and release of tissue transglutaminase, deaminated gliadin is presented to T-cells via HLA-DQ2 and HLA-DQ8 heterodimers, initiating enteropathy and further gut permeability through activated B-cells, pro-inflammatory cytokines, and cytotoxic T-cells (Fig. 3).^[50]^

Although this pathway has been clearly established, a significant question remains: what non-genetic factors may flip the switch from a genetic predisposition for the disease to active disease pathogenesis? The presence of HLA-DQ2 and HLA-DQ8 are the strongest genetic factors linked to the development of CD, and the risk of CD in individuals who lack the HLA-DQ2/8 heterodimers is low; however, it is clear that there are additional non-HLA genetic and non-genetic factors that contribute to CD, highlighting the complexity of CD pathogenesis.^[51]^ HLA-DQ2 and HLA-DQ8 prevalence in the general population is roughly 30% to 40%; however, only 3% of carriers are found to develop celiac disease.^[16]^ Carriers of HLA-DQ2 comprise 90% of patients with celiac disease, while carriers of HLA-DQ8 comprise the remaining 10% of individuals with the disease.^[18]^ The prevalence of HLA-DQ2 and HLA-DQ8 varies geographically.^[52]^ A higher prevalence of HLA-DQ2 has been reported in European populations; whereas, in the south Indian native general population HLA-DQ8 prevalence is higher than HLA-DQ2.^[53]^ Recent studies have suggested the rise in CD could be attributed to early life infections, antibiotic use, and dietary changes that affect the gut microbiome, highlighting the significant role that gut microbiota is proposed to play in CD pathogenesis.^[2,54]^ Further focusing on the importance of the gut microbiome in the pathogenesis of CD, pharmaceuticals, such as PPIs, that cause gut dysbiosis have been linked to the inflammatory response present in CD.^[55]^

In the past decade, PPI availability and use has increased; similarly, the prevalence and incidence of celiac disease has also continued to increase, with incidence rates increasing around 7.5% yearly in the Western world.^[56]^ The global prevalence of CD is 1.4%; however, further population-based studies are needed to determine the increased incidence of the disease annually.^[56,57]^ In a recent study analyzing the association between PPIs, the development of CD serology, and alterations in the gut microbiome, one participant in a cohort of 12 healthy participants taking omeprazole 40 mg twice daily for 4 to 8 weeks developed an increased immune reaction to deaminated gluten and CD autoantibody response to tissue transglutaminase (TG)2.^[2]^ While this was a very small study which limits the generalizability of the findings, others have linked gut dysbiosis with the adaptive immune response triggered in CD due to mucosal damage.^[55]^ Environmental factors contributing to CD, such as ingestion of gluten, early nutrition, composition of intestinal microbiota and antibiotics or other drug therapies, remain important issues in the development and progression of CD that warrant further investigation.^[58,59]^

Currently, a gluten-free diet is the standard treatment for patients with CD to reduce inflammation.^[60]^ However, recent evidence surrounding the mechanisms of advanced CD pathogenesis, such as IL-15 up-regulation in small intestine epithelium and gain-of-function mutations in janus kinase-signal transducer and activator of transcription pathway during the development of lymphomas as the result of impaired mucosal healing, reveals the potential for new therapeutic options to reduce the above mentioned adverse effect of CD.^[61]^ The challenge a strict gluten-free lifestyle places on patients with CD both socially and economically highlights not only the importance of finding new treatment options to improve the quality of life for individuals diagnosed with CD, but also the need to prevent the development of CD by controlling the interplay between environmental and genetic factors.^[62]^ In addition to potentially hastening the progression of CD, a recent observational study of 301 patients with newly diagnosed CD found that a gluten-free diet along with PPI exposure increased the risk of metabolic syndrome and hepatic stenosis development.^[63]^ Fortunately, there are several promising therapies, such as Nexvax2, a gluten vaccine, as well as HLA-DQ2 and HLA-DQ8 blockers in pre-clinical or clinical development. Advances in the treatment and management of CD are hopefully ongoing.^[64]^

## 4. Conclusion

PPI therapy is helpful in reducing acid production in gastroesophageal disorders such as gastroesophageal reflux, gastric ulcers, and BE; however, additional information is needed to determine whether PPIs are still an appropriate treatment option with the possibility of developing CD in the future, particularly in the context of HLA-DQ2 and HLA-DQ8 predispositions. The pathogenesis of celiac disease is multifactorial, and human leukocyte antigens are one factor that may contribute to its development. However, HLA-DQ2 and HLA-DQ8 are prominent factors that should be considered, especially in individuals with gastroesophageal conditions that require long-term PPI use. If prolonged PPI treatment causes more harm than benefit in certain individuals, both a new standard for prescribing PPIs and advanced alternative treatments will be necessary to avoid potential adverse effects.

## Author contributions

**Conceptualization:** Alexandra McMillan, Amanda E. Brooks.

**Writing – original draft:** Alexandra McMillan, Christopher Perez, Amanda E. Brooks.

**Writing – review & editing:** Alexandra McMillan, Amanda E. Brooks.
